# Subunit NDUFV3 is present in two distinct isoforms in mammalian complex I

**DOI:** 10.1016/j.bbabio.2016.12.001

**Published:** 2017-03

**Authors:** Hannah R. Bridges, Khairunnisa Mohammed, Michael E. Harbour, Judy Hirst

**Affiliations:** The Medical Research Council Mitochondrial Biology Unit, Wellcome Trust / MRC Building, Hills Road, Cambridge, CB2 0XY, U. K.

**Keywords:** Complex I, isoform, mitochondria, NADH:ubiquinone oxidoreductase, NDUFV3, rat

## Abstract

Complex I (NADH:ubiquinone oxidoreductase) is the first enzyme of the electron transport chain in mammalian mitochondria. Extensive proteomic and structural analyses of complex I from *Bos taurus* heart mitochondria have shown it comprises 45 subunits encoded on both the nuclear and mitochondrial genomes; 44 of them are different and one is present in two copies. The bovine heart enzyme has provided a model for studying the composition of complex I in other mammalian species, including humans, but the possibility of additional subunits or isoforms in other species or tissues has not been explored. Here, we describe characterization of the complexes I purified from five rat tissues and from a rat hepatoma cell line. We identify a ~ 50 kDa isoform of subunit NDUFV3, for which the canonical isoform is only ~ 10 kDa in size. We combine LC-MS and MALDI-TOF mass spectrometry data from two different purification methods (chromatography and immuno-purification) with information from blue native PAGE analyses to show the long isoform is present in the mature complex, but at substoichiometric levels. It is also present in complex I in cultured human cells. We describe evidence that the long isoform is more abundant in both the mitochondria and purified complexes from brain (relative to in heart, liver, kidney and skeletal muscle) and more abundant still in complex I in cultured cells. We propose that the long 50 kDa isoform competes with its canonical 10 kDa counterpart for a common binding site on the flavoprotein domain of complex I.

## Highlights

• Rat complex I contains the same 45 subunits as bovine complex I
• Complex I subunit NDUFV3 is present in two isoforms, a short and a long isoform
• The abundance of the long NDUFV3 isoform in complex I is tissue dependent
• The long NDUFV3 isoform is a ~ 50 kDa protein with unstructured regions

## 1 Introduction

Respiratory complex I (NADH:ubiquinone oxidoreductase) is the first enzyme of the electron transport chain in mammalian mitochondria [1]. It oxidizes NADH in the mitochondrial matrix to regenerate NAD^+^ and sustain crucial metabolic processes including the tricarboxylic acid cycle and β-oxidation of fatty acids, reduces ubiquinone in the inner membrane to supply electrons to respiratory complex III, and transports protons across the membrane, contributing to the proton motive force that drives ATP synthesis and transport processes. Complex I is also a significant source of mitochondrial reactive oxygen species production and so contributes to cellular oxidative stress. Due to its critical contribution to cellular metabolism, dysfunctions of complex I are the most frequent causes of mitochondrial disease [2]. Complex I defects are clinically and phenotypically diverse, and diagnosis of genetically-linked complex I dysfunctions, caused by mutations in both the mitochondrial and nuclear subunits of the enzyme, and in the assembly factors required for its biogenesis, relies on both biochemical and genetic information. Advances in high-throughput sequencing techniques have vastly increased the availability of genetic data in recent years [3], but interpretation of the links between specific mutations and clinical or pathological phenotypes still relies heavily on basic knowledge of the identities and sequences of the proteins involved.

Complex I isolated from *Bos taurus* (bovine) heart mitochondria is the most comprehensively studied mammalian complex I, and its subunit composition has been used as the model for the human enzyme. During the 1980s and 1990s Walker and coworkers identified 43 proteins in preparations of the bovine enzyme and its subcomplexes, and sequenced 35 different nuclear-encoded proteins [4], [5], [6]. Seven additional subunits were found to be encoded in the mitochondrial genome, making a total of 42 different sequences at this time [7]. The remaining protein was subsequently found to be an unusual fragment of one of the known subunits [8]. Then, in an extensive re-evaluation of the enzyme's subunit composition using state-of-the-art mass spectrometry methods, three more subunits were identified, giving a total of 45 different sequences [9], [10]. One of these proteins (NDUFA4), always in doubt as a *bona fide* subunit due to its weak association with complex I and its presence in more than one chromatographic fraction [11], has now been discounted as a complex I subunit [12]. The remaining 44 different subunits have been confirmed by determination of the structure of the bovine enzyme, and this also revealed that one subunit, the mitochondrial acyl-carrier protein, is present in two copies [13], [14]. Therefore, bovine heart complex I is currently understood to contain 45 subunits in total. Fourteen of the subunits of bovine complex I are the catalytic ‘core’ subunits that are conserved in all species of complex I and contain the mechanistic elements sufficient to catalyze NADH oxidation, ubiquinone reduction and proton translocation [1]. The additional 31 subunits present are ‘supernumerary’ or ‘accessory’ subunits [11]. They have been accumulated onto the core during evolution, and both their number and nature vary widely between species [15].

In general, the composition of complex I from other mammalian species has been assumed to be equivalent to that of the bovine enzyme. Extensive work to define the gene and protein sequences of the subunits of the human enzyme proceeded by identifying homologues to the bovine sequences [16]. Then, in 2003, Murray and coworkers used immuno-purified human complex I to detect 42 homologues to known bovine proteins [17] and, in 2005, Schilling and coworkers isolated complex I from mouse brain tissue and from cultured cells and detected 41 homologues to known bovine proteins [18]. In the same study complex I from a rat cell line was analyzed and 33 homologues were identified. Recently, complex I from ovine heart, a close relative of the bovine complex, has also been shown to contain the same 44 subunits [19]. However, all of these studies have taken the bovine heart complex as a model and not searched for species or tissue specific subunits or subunit isoforms that may not be present in it.

Here, we have characterized the composition of complex I isolated from five rat tissues: heart, skeletal muscle, kidney, liver and brain. We used chromatographic protocols developed for the bovine complex [20], adapted to small scale for the rat tissues, as well as immuno-purification from the same set of tissues and also cultured cells. The aims were to determine whether all 44 different subunits of the bovine enzyme are present in each rat tissue, and to search for additional proteins or isoforms that are associated with the complex, perhaps in a tissue-dependent fashion. We report discovery of a new isoform for subunit NDUFV3 that, with a molecular mass of ~ 50 kDa, is more than five times longer than the canonical ~ 10 kDa form.

## 2 Materials and methods

### 2.1 Cell culture

The MH-TC-5123 line was purchased from CLS Cell Lines Service GmbH, and the U2OS line (from Professor M. Ashcroft, Cambridge) was authenticated by Eurofins Genomics; all cells were confirmed negative for mycoplasma. MH-TC-5123 cells were cultured in RPMI medium (Gibco) supplemented with 4.5 g L^− 1^ glucose, 10% FBS (Hyclone) and 1 mM glutamine. U2OS cells were cultured in DMEM medium (Gibco), supplemented similarly.

### 2.2 Isolation of mitochondria and mitochondrial membranes from rat tissues

Mitochondria from the tissues of albino Wistar rats were prepared using protocols adapted from Chapel and Hansford [21] and Tyler and Gonze [22]. All the steps following tissue excision were at 4 °C. Briefly, the tissues were diced then homogenized. Skeletal muscle and heart were homogenized in buffer containing 30 mM Tris-HCl (pH 7.4), 225 mM mannitol, 75 mM sucrose and 0.5 mM EGTA by electrical homogenization, and brain, liver and kidney in 5 mM Tris-HCl (pH 7.4), 250 mM sucrose and 2 mM EDTA in a Dounce homogenizer. Debris was removed by centrifugation (1500 x *g* for 5 min) and by using a muslin cloth. Then, crude mitochondria were collected by centrifugation (10,000 x *g* for 25 min), resuspended in buffer containing 5 mM HEPES (pH 7.4), 250 mM mannitol and 0.5 mM EGTA, and layered onto 30% Percoll medium containing 25 mM HEPES (pH 7.4), 225 mM mannitol and 1 mM EGTA. The samples were centrifuged (95,000 x *g* for 30 min) and the mitochondria (in the lower brown band) collected and resuspended in buffer containing 20 mM Tris-HCl (pH 7.7), 10% glycerol and 1 mM EDTA. Membranes were prepared using a protocol adapted from that of Walker et al. [23] by addition of solid KCl to 0.15 M followed by electrical homogenization, and collected by centrifugation (13,500 x *g* for 40 min).

### 2.3 Isolation of mitochondria from U2OS cells

Mitochondria were isolated from cultured cells using a protocol adapted from that of Minczuk et al. [24]. U2OS cells (~ 1.5 g wet weight) were trypsinized from the flasks and washed twice in PBS. All subsequent steps were performed at 4 °C. The cells were resuspended in ~ 4.5 mL of buffer containing 20 mM HEPES (pH 7.8), 5 mM KCl, 1.5 mM MgCl_2_, 1 mg mL^− 1^ BSA and a protease inhibitor cocktail (Roche), incubated for 10 min, then disrupted by seven passes through a cell homogenizer (Isobiotec) fitted with a 12 μm ball. For every 3 mL of lysed cells, 2 mL of buffer containing 20 mM HEPES (pH 7.8), 525 mM mannitol, 175 mM sucrose, and the protease inhibitor cocktail were added, then the volume made up to 30 mL with MSH buffer (containing 210 mM mannitol, 70 mM sucrose, 20 mM HEPES (pH 7.8), 2 mM EDTA and the protease inhibitor cocktail). Cell nuclei were removed by centrifugation (750 x *g* for 10 min), then crude mitochondria were collected (8000 x g for 20 min). They were resuspended in MSH, treated for 15 min with 50 U/mL benzonase (Millipore), then layered onto a 1.5–1–0.5 M sucrose step gradient (also containing 10 mM HEPES (pH 7.8) and 5 mM EDTA). Following centrifugation (8500 x *g* for 60 min), the brown band was collected, diluted in MSH buffer, and recentrifuged to collect the mitochondria.

### 2.4 Purification of complex I by chromatography

The following method was adapted from that of Sharpley et al. [20]. Mitochondrial membranes (~ 5 mg mL^− 1^) were solubilized by addition of 0.8–1.2% lauryl maltose neopentyl glycol (LMNG, Anatrace) for 30 min. Insoluble materials were removed by centrifugation (4800 x *g* for 30 min) then the supernatant was filtered (0.22 μm polyethersulfone syringe filter) and loaded onto a Q-sepharose HP column (GE Healthcare) pre-equilibrated with buffer A (20 mM Tris-HCl (pH 7.7), 10% ethylene glycol, 0.1% LMNG, 1 mM EDTA, 0.005% soy bean asolectin (Avanti Polar Lipids) and 0.005% CHAPS (Santa Cruz Biotechnology). The column was washed with 22 or 24% buffer B (buffer A plus 1 M NaCl) then complex I was eluted at 33 or 35% buffer B using either a linear or step elution. To remove F_1_F_o_-ATPase, complex-I containing fractions were incubated for 15 min with 1 mM ATP, 1 mM MgSO_4_ and 0.25 μg of a modified version of the bovine ATPase inhibitor protein IF_1_, carrying a C-terminal histidine tag [25]. The samples were passed through a HisTrap HP column (GE Healthcare), pre-equilibrated with buffer containing 20 mM Tris-HCl (pH 7.7), 10% ethylene glycol, 0.1% LMNG, 1 mM EDTA and 20 mM NaCl, collected and concentrated. Finally, the sample was applied to a 2.4 mL Superose 6 size exclusion column run in buffer containing 20 mM Tris-HCl (pH 7.7), 10% ethylene glycol, 0.02% LMNG, and 150 mM NaCl; the complex I-containing fractions (which eluted at ~ 1.3 mL) were collected and concentrated for storage at − 80 °C.

### 2.5 Purification of complex I by immuno-purification

Complex I was immuno-purified from rat tissue mitochondria using a commercial kit (Abcam ab-109,711) according to the manufacturers instructions, and from cell line MH-TC-5123 by the same method but following the digitonin treatment of Andrews et al. [26]. All steps were carried out at 4 °C. Briefly, mitochondria and digitonin-treated cells were solubilized with 1% *n*-dodecyl β-D-maltoside (DDM), centrifuged to remove insoluble material, and incubated with the affinity beads overnight. The beads were washed twice before complex I was eluted in buffer containing 200 mM glycine (pH 2.5) and 0.05% DDM. The pH was neutralized by addition of Tris base.

### 2.6 Electrophoresis

Samples were reduced with dithiothreitol or tris(2-carboxyethyl)phosphine and analyzed by SDS PAGE using tris-glycine 10–20% acrylamide gels (Life Technologies) with bovine complex I and a molecular weight ladder (Kaleidoscope, Bio-Rad) for comparison. Mitochondria from U2OS cells were analyzed by Blue Native PAGE (3–12% acrylamide gels, Life Technologies) at 4 °C according to the manufacturers instructions; after 1 h the standard cathode buffer was replaced with one containing 10% of the Coomassie concentration. All gels were stained with Coomassie R-250.

### 2.7 Creation of a custom database of rat complex I subunit sequences

A custom sequence database, based on the Ensembl (http://www.ensembl.org) database Rnor_6.0.all (July 2015), was created in order to search mass spectra generated from rat proteins. All the sequences for complex I subunits were annotated with meaningful descriptions, and aligned with their well-characterized bovine counterparts to inspect their presence, their sequence homologies and their lengths. Sequences for rat subunits ND2 and NDUFS6 were absent from the downloaded resource so, respectively, NCBI sequences (https://www.ncbi.nlm.nih.gov/protein) AP_004893.1 and NP_062096.1 were added. The sequence for rat subunit NDUFB1 was also absent, but the only candidate replacement protein sequences in the NCBI database were derived from low quality DNA sequences and hence contained undetermined residues. Therefore, the well-characterized bovine sequence was used to search the rat chromosomal sequences with the tblastn application (https://blast.ncbi.nlm.nih.gov/Blast.cgi?PROGRAM=tblastn). Two short regions of homologous protein were identified in different reading frames on chromosome 6. The chromosomal sequence covering both regions was examined using the Augustus gene prediction program [27], and the predicted protein sequence (found to be identical to EST sequence AI170469.1 from a *R. norvegicus* lung sample) was added to the database.

The custom database was further augmented with the predicted mRNA splice variants of complex I subunit-encoding genes. Sequences were retrieved from the NCBI Aceview mRNA resource [28] and the Augustus application [27] was used to predict further hypothetical isoform variants from portions of the chromosomal DNA sequences for each gene from Rnor_6.0, extended with 10 kbp of the flanking regions. The procedure was carried out using the parameters for predicting human splice variants. In total, 182 alternative isoforms for 34 subunits were added to the database; no alternative transcripts were predicted for subunits NDUFA2, NDUFA13 or NDUFB10. The custom database was uploaded to an in-house Mascot 2.4 server (Matrix Science Ltd.)

### 2.8 Mass spectrometry analyses

Protein samples for mass spectrometry analyses were either precipitated from solution with cold ethanol and digested in 50 mM ammonium bicarbonate with trypsin, or digested in-gel in 20 mM Tris-HCl (pH 8) and 5 mM CaCl_2_, with subsequent peptide extraction. For LC-MS analyses, samples were fractionated on an Acclaim PepMap nanoViper C18 reverse-phase column (Thermo Scientific) (75 μm × 150 mm), with a gradient of 5–40% acetonitrile in 0.1% formic acid at a flow rate of 300 nL min^− 1^ over 84 min, then peptides were analyzed by a Q-Exactive Plus Orbitrap mass spectrometer (Thermo Scientific) with fragmentation performed by higher-energy collisional dissociation (HCD) using nitrogen. The mass range was 400 to 1600 *m*/*z* for the precursor ions; the top 10 most abundant ions were selected for MS/MS analyses. One sample, the in-solution tryptic digest of a chromatographically purified complex I from liver, was analyzed with an LTQ Orbitrap XL mass spectrometer by the closely-related method described by Andrews et al. [26]. Spectra were assigned to peptide sequences and originating proteins using the Mascot 2.4 application (Matrix Science Ltd.) with a peptide precursor mass tolerance of 5 ppm and fragment mass tolerance of 0.01 Da (0.5 Da for data from the Orbitrap XL), allowing for up to four missed cleavages and variable modifications (methionine oxidation, protein *N*-formylation and *N*-acetylation). For analysis of peptides extracted from SDS PAGE gel slices by matrix-assisted laser-desorption ionization (MALDI), an Applied Biosystems/MDS SCIEX model 4800 Plus MALDI–TOF-TOF spectrometer was used [29]. Spectra were assigned using the same variable modifications plus cysteine propionamide and allowing for up to three missed cleavages, with a peptide mass tolerance of 360 ppm and a fragment mass tolerance of 0.8 Da. Only peptide assignments made by Mascot scoring at or above its *P* < 0.05 threshold were considered.

### 2.9 Blue native PAGE profiling, with automated relative peptide intensity analyses

Profile analyses of mitochondria from U2OS cells were carried out following the method of Heide et al. [30]. Equally-sized slices from a BN-PAGE lane were digested with trypsin, peptides extracted by addition of 60% acetonitrile, 4% formic acid, and a portion of each extraction dried down to completion and resuspended in 2% acetonitrile, 0.1% formic acid. The peptides in each slice were analyzed sequentially using the Q-Exactive Orbitrap Plus instrument as described above. For peptide sequence assignment, ProteomeDiscoverer (Thermo Fisher Scientific) software was used to submit spectra to Mascot 2.4 (Matrix Science Ltd.) configured to use a Uniprot (http://www.uniprot.org) *Homo sapiens* database (UP00005640, December 2015), augmented with the sequence of the long isoform of NDUFV3 (termed NDUFV3L). Search parameters included the same variable peptide modifications as above, with cysteine propionamide and allowing for the presence of only one missed cleavage, and a peptide mass tolerance of 10 ppm and fragment ion mass tolerance of 0.5 Da. ProteomeDiscoverer counted the abundances for all the well-assigned peptides observed in all the samples, and its standard aggregation algorithm was employed as a measure of relative protein abundance for each protein for each gel slice; up to the three highest intensity peptides for each protein were selected and averaged.

### 2.10 Semi-automated relative peptide intensity analyses

The raw mass spectra from LC-MS analyses were analyzed in greater detail specifically for evidence pertaining to the long and short isoforms of NDUFV3. Xcalibur Qual Browser (version 3.0.63, Thermo Fisher Scientific) was used to create extracted ion chromatograms (XICs) for peptides identified previously with Mascot in various ion charge states. XICs were created and inspected for the 2^+^, 3^+^ or 4^+^ charge states of various ions from complex I subunit peptides, with mass tolerances of 20 ppm. The peak volume observed at the expected retention time of the peptide was calculated using Xcalibur's integration tool, and the fragmentation spectrum of the peptide was examined to validate its identity. The sums of the intensities for peptides unique to the long and short isoforms were normalized to the sum of the intensities for the common peptides, to allow comparison between samples.

### 2.11 Bioinformatic analyses

Homologues to the human sequences for the long and short isoforms of NDUFV3 were identified by tBlastn searches against the NCBI RNA RefSeq database using the BLOSUM62 scoring matrix. Sequences that had an expect value of < 10^− 4^ were considered to be homologous. Secondary structure analyses were carried out using PSIpred [31] and PredictProtein [32], and GlobPlot 2.3 [33], Disprot [34], and DisEMBL [35] were used to search for regions of intrinsic protein disorder. Sequence motifs were investigated using NCBI-CDD [36] and Pfam [37]. *N*-terminal cleavage sites for mitochondrial import sequences were predicted by using Mitoprot [38], TargetP [39], and by comparison to the known bovine cleavage sites. Canonical R-2, R-3 or R-10 motifs for mitochondrial processing peptidase (MPP) cleavage [40] were detected for all subunits with cleaved import sequences except for NDUFS7, NDUFS8, NDUFA10 and NDUFV3.

## 3 Results

### 3.1 Purification of complex I from five rat tissues

**Fig. 1:**
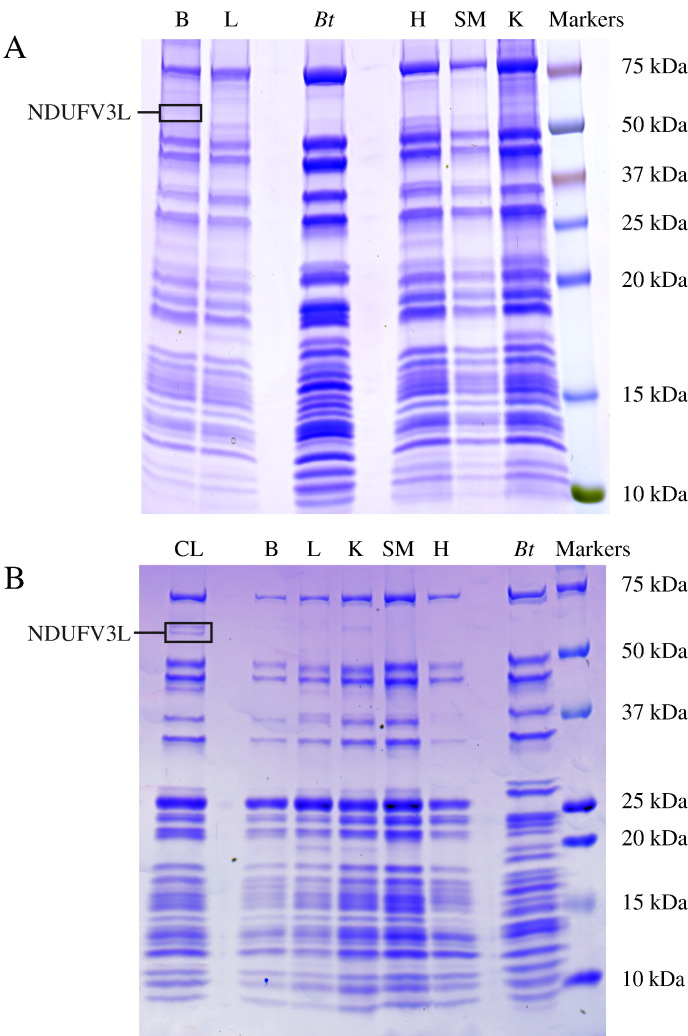
**SDS PAGE analyses of isolated rat complex I preparations.** A) Samples purified by chromatography. B) Samples purified by immuno-purification. The boxes show the gel regions where NDUFV3L was detected by MALDI-TOF analyses. B, brain; L, liver; H, heart; SM, skeletal muscle; K, kidney; *Bt*, complex I isolated from bovine heart; CL, sample from the rat MH-TC-5123 cell line.

Complex I was isolated from mitochondrial membranes prepared from five different rat tissues (heart, skeletal muscle, liver, kidney and brain) by adapting to a smaller scale chromatographic methods established for complex I from bovine heart mitochondria [20]. In general, the rat enzymes were less stable than the bovine enzyme, being more prone to aggregation upon solubilization, and the resulting preparations were less pure; modifications made to address these problems included the use of a different detergent, lauryl maltose neopentyl glycol (LMNG) for soubilization and chromatography, and inclusion of an affinity step to remove ATP synthase. Fig. 1A shows a typical SDS PAGE analysis of the complex I samples isolated from each tissue, and compares them to a sample of the bovine heart enzyme. It is clear from the banding pattern that the subunit composition is similar in all cases, although the positions of some of the bands vary between the species, and a higher level of contamination is present in the rat samples, particularly the brain and kidney samples. Complex I was also isolated from the same tissues by immuno-purification, using a commercial kit (Fig. 1B). Again, the banding patterns for all five samples are similar, but clear extra bands are apparent in the immuno-purified samples; the strong band at ~ 25 kDa originates from the antibodies used in the affinity kit, and others are due to non-complex I proteins that bind adventitiously to it. For comparison with the enzyme from tissues, the complex was also isolated from the rat hepatoma cell line MH-TC-5123 by immuno-purification.

### 3.2 Identification of known mammalian complex I subunits in the rat complexes

**Fig. 2:**
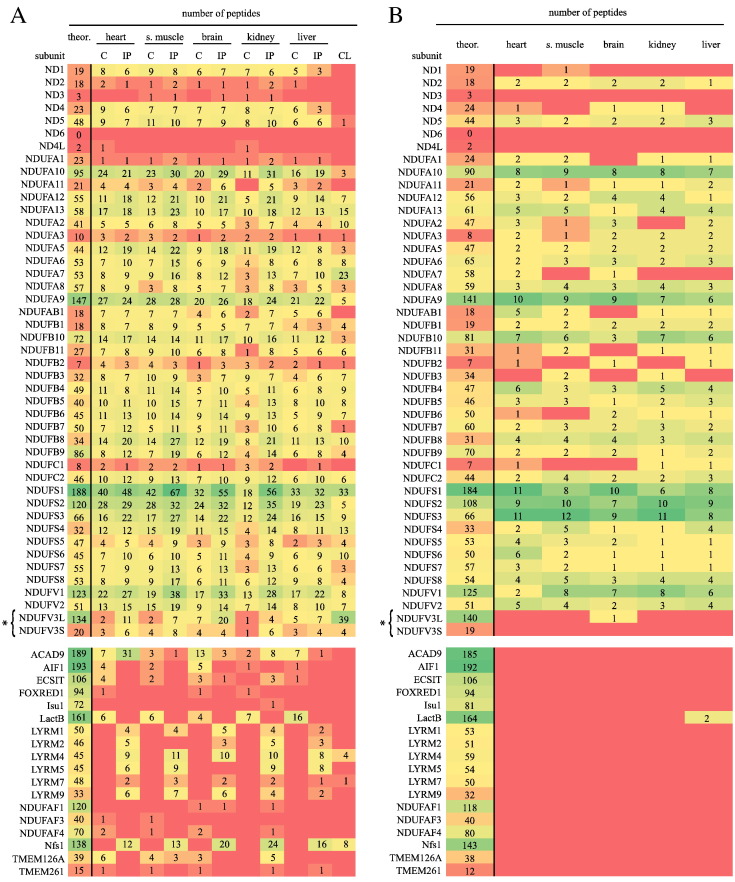
**Heatmap summary of the identification of complex I subunits.** The number of theoretical tryptic peptides for each subunit (within the measured mass range) are presented alongside the number detected in each sample. A) summarizes LC-MS data from unfractionated digests and B) summarizes MALDI-TOF data obtained following SDS PAGE fractionation. C, chromatographically purified sample; IP, immuno-purified sample; CL, sample from the rat MH-TC-5123 cell line.

Each of the rat complexes were analyzed by mass spectrometry to detect the presence of homologues to the 44 different subunits present in the highly-characterized enzyme from bovine heart [5], [8], [11], [41]. First, samples of chromatographically- and immuno-purified complex I from all five tissues were digested in solution with trypsin, and the peptides were analyzed by LC-MS. The results are summarized in Fig. 2A, and presented in full in Supplementary Tables 1 and 2. In addition, the complete mass spectrometry data are given in Supplementary Dataset 1. For the chromatographically-purified complexes, homologues of 42 bovine subunits were detected in the samples from heart and skeletal muscle, 41 in the samples from liver and brain, and 43 in the sample from kidney. For the immuno-purified complexes, 41 subunits were detected in the sample from heart, 42 in the samples from skeletal muscle, brain and kidney, and 40 in the sample from liver. The detection of any subunit relied on at least one peptide scoring above the Mascot 95% confidence threshold. Fig. 2A uses a heatmap to compare the number of peptides observed for each subunit with the number of tryptic peptide ions with 2^+^ and 3^+^ charges predicted by the PeptideMass application [42] to be possible within the mass range (400–1600 *m*/*z*), and allowing for up to four missed cleavages. Although the seven highly-hydrophobic mitochondrial-encoded subunits were not all detected in all samples, they are core components of the enzyme complex and their presence in the enzyme is not in doubt. The presence of every one of the 30 known supernumerary subunits in each of the five tissues was established, with only two missed identifications (NDUFC1 in the chromatographically-purified liver enzyme, and NDUFA11 in the chromatographically-purified kidney enzyme) of small subunits with relatively few tryptic peptides. The immuno-purified complex from the MH-TC-5123 cancer cell line was analyzed similarly and 34 subunits were detected. In addition to six of the hydrophobic ND subunits, subunits NDUFA1, NDUFA11, NDUFAB1 and NDUFC1 were not detected.

Peptides characterized from the rat complex I samples were sufficient to define the *N*-terminal modifications and the mitochondrial target peptide cleavage sites for 31 subunits. Table 1 summarizes the *N*-terminal modifications observed here (the complete data are given in Supplementary Table 3), alongside those determined previously in the highly-characterized bovine enzyme [11], [43]. Only one minimal difference was identified: for rat NDUFB1, proteins both with and without the initiator methionine were detected. On this basis it is likely that the *N*-terminal processing of the remaining 13 rat subunits also match their bovine homologues: the predicted modifications and cleavage sites are included in Table 1.

### 3.3 Additional proteins detected in the rat complex I samples

In order to investigate the possibility that extra species- or tissue-specific subunits are present in one or more of the rat complexes when compared with the highly-characterized complex from bovine heart, the additional proteins detected by LC-MS mass spectrometry were considered. Many of them, such as subunit-α of F_1_-ATP synthase, have well-characterized alternative known functions and are common contaminants of complex I preparations; we do not assign a biological significance to their presence. However, a considerable number of other proteins were also detected (Supplementary Dataset 1). Here, we focus only on those proteins (see lower panels of Fig. 2) that have been associated with complex I previously. Table 2 shows that only ACAD9, ECSIT and TMEM126A, proteins involved in the assembly of complex I [44], were detected in both chromatographically- and immuno-purified samples. AIF1 (which has recently been linked to the MIA40 pathway for oxidative folding of the three CHCH domain-containing subunits of complex I [45], [46]) and FOXRED1, NDUFAF3 and NDUFAF4 (which are also complex I assembly factors [44]) were detected in only chromatographically-purified samples, along with mitochondrial lactamase-β (lactB), which has been linked to complex I by phylogenetic profiling [47]. NDUFAF1 (a further complex I assembly factor [44]), Isu1 and Nfs1 (involved in the assembly of iron-sulfur clusters [48]) and six LYR proteins were detected only in immuno-purified samples; the LYR proteins share an amino-acid motif with subunits NDUFB9 and NDUFA6 [49], [50] so it is likely that they interact directly with the immunoaffinity matrix and are artificially enriched. As contaminant proteins are less likely to be present in both chromatographically- and immuno-purified samples, ACAD9, ACADVL, ECSIT and TMEM126A are the most likely candidates to interact with the mature complex.

To investigate these proteins more thoroughly two additional approaches were used. First, the LC-MS analyses described are highly sensitive and detect many proteins present at extremely low levels: if a protein can also be identified by MALDI-TOF mass spectrometry, a relatively less sensitive technique with lower dynamic range, then it is likely present in a more substantial amount. The chromatographically-purified samples (containing the most candidate proteins) were resolved by SDS PAGE, each gel lane was cut into 50–60 slices, then the proteins present in each section were analyzed by MALDI-TOF (the compositions of unfractionated digests of the complex are too complicated for meaningful MALDI-TOF analysis). Fig. 2B summarizes the MALDI-TOF data, for comparison with the LC-MS data in Fig. 2A. A substantial number of the known subunits were detected (37 in the sample from heart, 34 in the sample from skeletal muscle, and 35 in the samples from liver, kidney and brain, all detected in gel sections consistent with their masses) but the only complex I-associated protein detected was lactB in the sample purified from liver. Second, we interrogated existing blue-native PAGE profiling data on mitochondria from the human osteosarcoma cell line U2OS, to search for proteins that co-migrate with complex I. None of the proteins investigated showed a distinct intensity peak co-incident with mature complex I. In summary, it is highly unlikely that any of the additional proteins detected are specifically associated with mature complex I in any of the cells or tissues studied, suggesting they are simply low-level contaminants or they are present in partially-assembled intermediates.

### 3.4 Identification of an alternative isoform of subunit NDUFV3 in complex I (NDUFV3L)

**Fig. 3:**
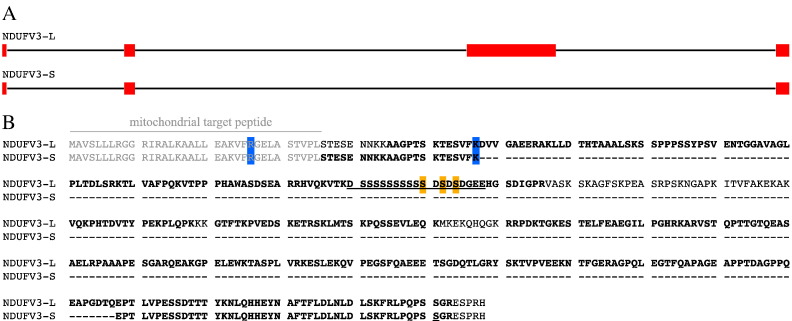
**The two isoforms of NDUFV3.** A) The gene structure of NDUFV3 in *R. norvegicus* with the exons shown in red and the introns as black lines. B) Pairwise sequence alignment of the two protein products. The mitochondrial targeting peptide is in grey and two residues that were identified as variants in mitochondrial disease patients [3] and conserved between the human and rat sequences are marked in blue. Residues in bold were detected by peptide mass fingerprinting, and serine residues homologous to phosphorylated residues identified in proteomics studies are marked in orange [57], [58], [59]. An unusual acidic region featuring a chain of serine residues in the same region is underlined.

To search for alternative splice variants of the complex I subunits, two isoform prediction programs were used to construct the protein sequences of hypothetical alternative isoforms (a total of 182 isoforms were predicted for 34 subunits). These sequences were added to the database of rat protein sequences, and compared with the LC-MS dataset. Discerning between isoforms present on the basis of peptide data is complicated by the fact that many peptides are common to multiple isoforms. Thus, identifications of isoforms made solely on the basis of one or more peptides also present in the canonical isoform were discarded. Only one splice variant was unambiguously identified: a ‘long’ isoform of subunit NDUFV3 (NDUFV3L). The canonical ‘short’ NDUFV3 subunit (NDUFV3S) has a mass of 8.2 kDa and is generally referred to as the 10 kDa subunit. The long isoform has a mass of 45.6 kDa and so, by analogy, we refer to it as the 50 kDa isoform. Fig. 3 compares the gene structure and protein sequences for the two isoforms; exons 1 and 4 are common to both, but exon 3 is only expressed in the long version. No evidence for an alternative isoform of NDUFB11, as detected previously in cultured human cells [51], was identified.

Peptide evidence obtained for both NDUFV3 isoforms is summarized in Table 2. In LC-MS analyses of the chromatographically-purified samples, peptides unique to the long form of NDUFV3 were observed only in complex I from liver and brain, whereas peptides unique to the short isoform, or common to both, were observed in the samples from all tissues. In LC-MS analyses of the immuno-purified samples, peptides unique to both the short and long isoforms, as well as those common to both, were observed in all samples. Thus, the long isoform was detected in complex I samples purified by both methods. Considerably more peptides derived from the long isoform were detected in the sample isolated from cultured cells. Although the number of peptides detected is not a reliable quantitative measure of protein abundance, the data suggest that the long isoform is present at higher abundances in brain and liver, and that it is significantly upregulated in cultured cells. In MALDI-TOF analyses, which as mentioned are much less sensitive, the long isoform was detected only in chromatographically-purified complex I from brain, and numerous peptides were detected in the sample from cultured cells (see Table 2). Crucially, in both cases the peptides originated from the ~ 55–60 kDa region of the SDS PAGE analysis (see Fig. 1) where a band is visible in the lane containing the sample from cultured cells. In support of a higher level of the long isoform in brain than in the other tissues, LC-MS analyses of the same ~ 55–60 kDa region from SDS PAGE analyses of mitochondria isolated from each tissue detected the long isoform only in the sample from brain.

**Fig. 4:**
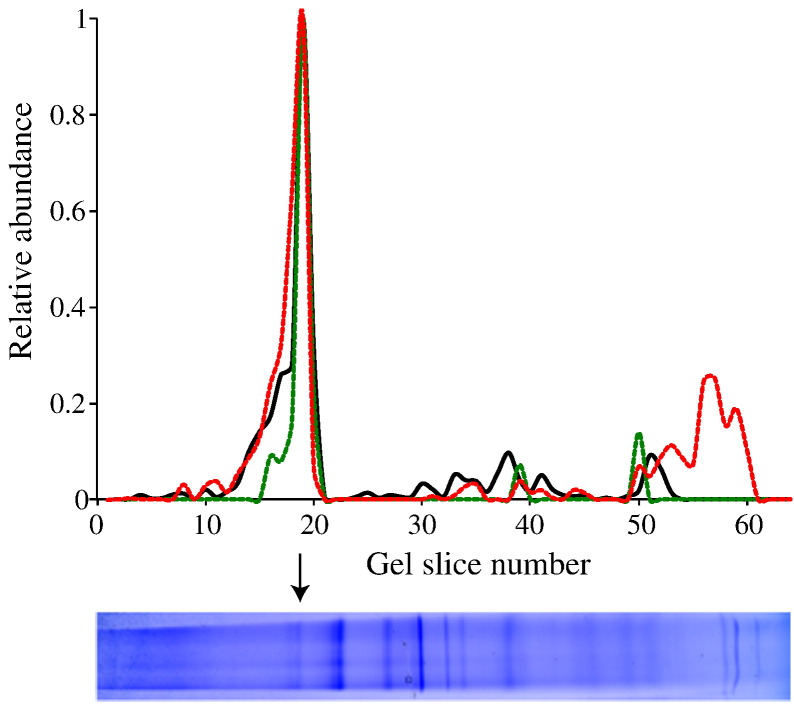
**Blue native PAGE profiling for the NDUFV1, NDUFV3L and NDUFV3S subunits of complex I**. The gel slice shown was cut into approximately 60 slices and each slice analyzed by LC-MS. The signal intensities for the different subunits (black for NDUFV1, red for NDUFV3L and green for NDUFV3S) are plotted against the gel, showing that all three proteins have their greatest abundance in the same slice, corresponding to the position for intact complex I. Note that the slice width is not precise so the graph scale and gel do not match exactly.

Finally, Fig. 4 shows the relative intensity profiles for NDUFV1, NDUFV3 and NDUFV3L in a blue native PAGE analysis of mitochondria from human U2OS cells. The peak for NDUFV1 (a core subunit of complex I) in slice 19 corresponds to the position of mature complex I, and therefore it is clear that both the long and short isoforms of NDUFV3 are associated with the intact complex. A proportion of NDUFV3L is also present towards the bottom of the gel, in a position corresponding to the monomeric protein. NDUFV3 is located peripherally in the molecular structure of bovine complex I [14], so it may be only weakly bound and dissociate during electrophoresis – or it may also be present as the free protein in mitochondria. In summary, our data suggest that NDUFV3L is a *bona-fide* component of rat complex I, and present also in the human enzyme, but that it is present in a substoichiometric level that varies between tissues.

### 3.5 Estimation of the relative abundance of the long and short isoforms in different tissues

**Fig. 5:**
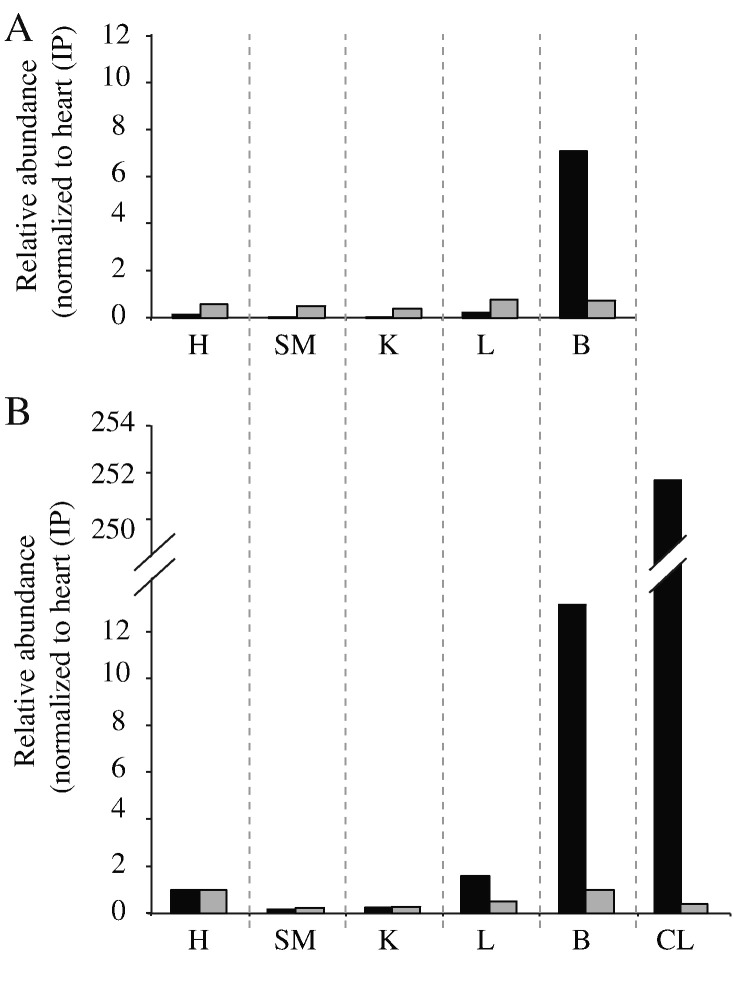
**Unlabeled peptide quantification of NDUFV3S and NDUFV3L.** A) Chromatographically- purified complexes I. B) Immuno-purified complexes I. The short isoform is in grey and the long isoform in black. H, heart; SM, skeletal muscle; K, kidney; L, liver; B, brain: CL, rat MH-TC-5123 cell line.

The relative abundance of the long and short isoforms of NDUFV3 in different tissues was evaluated in a low-precision estimation based on unlabeled peptide signal intensities from LC-MS analyses. Two peptides unique to the long isoform, two unique to the short isoform, and two common to both isoforms were selected from the sets of peptides that surpassed the 95% Mascot confidence threshold in at least one sample (see Supplementary Table 4). A total of 110 peptide intensities (including multiply charged ions) were manually assessed in the 11 samples investigated, and 100 peaks were quantified. For 73 of them the fragmentation patterns were matched to the sequence by Mascot, confirming the peptide identities. For six of them the fragmentation patterns were not matched but were similar to those matched by Mascot, and the remaining 21 assignments were based on shared peptide masses and retention time only. The peak volumes for the two peptides unique to the long isoform were summed, as were those for the common and short-isoform peptides. To account for variation in sample composition, the relative abundance estimate for the long isoform was obtained by dividing the unique-to-long-isoform summed value by that of the common-to-both summed value, and this calculation was made similarly for the short isoform. Then, to provide an estimation of how the relative abundance of each isoform varies between tissues, each value was normalized to the value from immuno-purified heart complex I. The values obtained are given in Fig. 5. Note that they provide no information about absolute stoichiometries, only a comparison of the relative amounts in the different tissues. Consistent with the observations described above, our data suggest that the long isoform is considerably more abundant, relative to the short isoform, in complex I from cultured cells, and also more abundant (though to a lesser extent) in complex I from brain tissue. The relative abundance appears to be low (and similar) in all other tissues; the lack of precision in the estimated values precludes further interpretation.

## 4 Discussion

### 4.1 The canonical mammalian complex I

45 subunits were identified and modeled in the structure of complex I from *B. taurus* heart mitochondria [13], [14], [52], substantiating the consensus value of 44 different subunits and two copies of the acyl-carrier protein determined by protein and mass spectrometry analyses [5], [8], [11], [41]. The subsequent structure of ovine complex I showed the same set of 45 subunits [19], whereas the transmembrane helix from subunit NDUFB3 (as well as subunit NDUFA12 on the hydrophilic arm) were not modeled in the complex I-containing structure of the porcine respirasome [53]. Previous MALDI-TOF analyses of the complexes I from ovine and porcine hearts (prepared using the method of Sharpley and coworkers) [54] detected all 30 expected supernumerary proteins in the porcine complex, and only missed identifying subunit NDUFC1 in the ovine complex, so it is likely that these two subunits are present in the porcine complex, but that their density is only poorly resolved. Notably, the subunit compositions of the *B. taurus*, *S. scrofa* and *O. aries* complexes were all determined using intact and functional chromatographically-purified material. Previously, mass spectrometry analyses of the human and rodent complexes [17], [18] have been performed on immuno-purified material, and on the rodent enzyme purified using a sucrose gradient, and the majority of the expected subunits were detected. Here, we have identified the complete set of 30 supernumerary subunits in intact chromatographically-purified rat complex I (a mammalian species less closely related to *B. taurus* than *S. scrofa* and *O. aries*). Together, these analyses confirm the composition of the canonical 45-subunit mammalian complex I, supporting different mammalian species being used as models for the human enzyme. Furthermore, identification of the same 44 subunits in five different rat tissues (and in cell lines) suggests that the canonical enzyme is not specific to heart tissue, and identification of the same *N*-terminal modifications in the rat and bovine enzymes suggests that they are also common throughout mammals.

### 4.2 The challenge of identifying hitherto-unknown subunits

Identifying the presence of known subunits in a sample of complex I is relatively straightforward, provided that data are searched against a sufficiently complete and well annotated database. Evaluating whether additional subunits are present is a much greater challenge: many additional proteins are detected in mass spectrometry analyses but it is difficult to decide if they are contaminants, proteins that bind transiently such as assembly factors, chaperones, and proteins involved in signaling or degradation pathways — or subunits. Here, we investigated additional proteins in our preparations by discarding proteins with known alternative functions, comparing data from different methods of isolation and analyses, and by focusing on proteins with a previous reported connection to the complex. We did not identify any new candidate subunits, in any of the tissues considered — but cannot exclude the possibility that tissue-specific subunits exist to be identified in the future.

### 4.3 The ‘long’ isoform of subunit NDUFV3 in complex I

The long, 50 kDa isoform of NDUFV3 has been found to be associated with complex I in five different rat tissues, and in both rat and human cultured cells, including in complex I samples isolated by three different methods (chromatography, immuno-purification and blue native PAGE). The long isoform is most abundant in brain tissue, and particularly abundant in cultured cells, and the very low levels detected in heart, skeletal muscle, kidney and liver are consistent with the fact that it has never been detected in the extensively characterized enzyme from bovine heart. However, even in cultured cells (with the highest abundance of NDUFV3L) only a faint band is visible in SDS PAGE analyses (Fig. 1), showing that the long isoform is substoichiometric in all cases, particularly so in heart, skeletal muscle and kidney.

Fig. 3 shows that the long, 50 kDa isoform of NDUFV3 shares its N- and C-termini with the short, 10 kDa (canonical) form, but contains a large insertion between them. Bioinformatic analyses suggest that a large portion (predicted 40–90%, depending on the analysis program used) of the protein is present as disordered loop structures, no substantial secondary structural elements are predicted to be present, and no strong homology to any other protein can be identified (although motif searches suggested some similarity to ribonuclease E, DNA polymerase II subunits γ and τ, and DNA topoisomerase 2). A notable feature of the structure of the hydrophilic domain of complex I from *B. taurus* [14] are the long loops that run across its surface, particularly at subunit interfaces. A good example is subunit NDUFA7, which arches up from the bottom of the domain along the NDUFS8–NDUFS2 subunit interface, crosses NDUFS2, runs along its interface with NDUFS1 and then onto NDUFS3. A long loop from the central portion of NDUFV3 would thus be consistent with the structures and forms of other subunits on the same domain. In the structure of complex I from *B. taurus*, NDUFV3 is located at the top of the hydrophilic arm, adjacent to NDUFV1 and NDUFV2 [14], most likely with the conserved C-terminal region in a cleft between NDUFV1 and NDUFV2. It is thus likely that the *N*- and *C*-termini common to both isoforms anchor the subunit to the complex, and that the two isoforms are interchangeable. Stroud and coworkers [55] suggested that in cell lines NDUFV3 is the final subunit to assemble into the mature complex, and demonstrated that copies of the short isoform are readily exchanged in and out of the mature complex *in vivo*. Consequently, we propose that the long and short isoforms compete for a common binding site on complex I (such that each complex contains a single NDUFV3 subunit of either isoform), with their relative levels of incorporation in different cells and tissues governed by the relative levels of the isoforms present in the mitochondria.

### 4.4 Conservation of NDUFV3L in other species

**Fig. 6:**
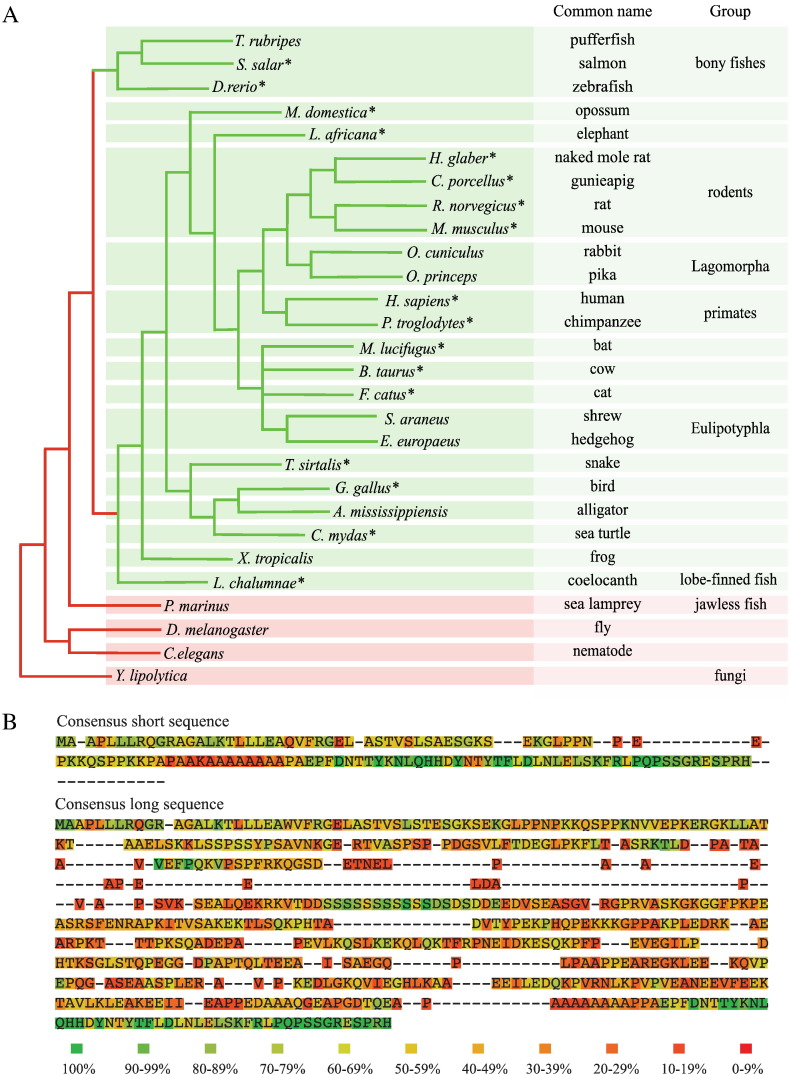
**The NDUFV3 isoforms in different species.** A) Phylogenetic tree showing the distribution of the two NDUFV3 isoforms. Species in red boxes do not contain NDUFV3. Species in green boxes do contain NDUFV3 and an asterisk is used to denote those for which the long isoform has been identified. B) Consensus sequences for the two isoforms based on multiple sequences alignments from the species in A. The most common residue at each position is given, and colors indicate the percentage identity. A possible *N*-terminal extension to the short sequence from *Xenopus tropicalis* has been omitted as a methionine is also present at the usual position of the start codon; the length of the alignment is extended by insertions in the NDUFV3L sequence from salmon.

To investigate the wider prevalence of the NDUFV3 splice variants, the NDUFV3L and NDUFV3S sequences were searched against the NCBI RNA database (see Fig. 6). NDUFV3 appeared in evolution around the time that lobe-finned fishes developed, as a homologue could not be identified in jawless fish, insects, nematodes or fungi. Analysis of the structure of the human NDUFV3 gene revealed the same splicing pattern as in rat (consistent with detection of NDUFV3L in human U2OS cells) and the alternative splicing is known to involve the catenin protein known as ‘Armadillo Repeat gene deleted in Velo-Cardio-Facial syndrome’ (ARVCF) [56]. NDUFV3L itself is present in many species from fish to humans — and it is possible that for some species RNA sequencing has not yet been deep enough to identify it. Sequence alignments for the NDUFV3 homologues identified (see Fig. 6) show that both the C-terminus (conserved in both isoforms) and an unusual region containing a string of ten serine residues (see also Fig. 3), particular to the long isoform, are well conserved, but that the rest of the protein sequence is only poorly conserved.

### 4.5 The role of NDUFV3L

Very little information pertaining to a physiological role for NDUFV3L is available. First, four mutations in NDUFV3 have been observed in patients with mitochondrial disease [3] (see Fig. 3), although they have not been confirmed as causatory. Two of the residues are conserved in rat (R26Q and K56N). R26 is part of the mitochondrial import sequence, so the mutation may affect mitochondrial import and *N*-terminal processing. The codon for K56 is adjacent to the exon2-intron2 boundary, however the mutation is not predicted to affect splicing, and its presence at ~ 0.5% in the ExAC database (http://exac.broadinstitute.org), including as a homozygous variant, suggests it is not pathogenic. The two non-conserved residues are in sequence that is specific to the long isoform. Second, phospho-proteome analyses [57], [58], [59] have identified three of the conserved serine residues in the central serine-rich motif to be phosphorylated in various conditions (see Fig. 3) – and indeed NDUFV3L was first discovered as a 65 kDa mitochondrial phosphoprotein (MIPP65) in rat tissue, as a substrate for protein kinase N1 [60]. Third, NDUFV3 is located on chromosome 21 and so is upregulated in patients with trisomy 21 Downs syndrome [61]. Fourth, NDUFV3L has been found to be up-regulated in cortical ischemia [62] and this, in conjunction with our finding of a higher level of NDUFV3L in brain complex I, may suggest it has a tissue-specific role. Finally, in its size, lack of secondary structure, and proximity to the flavin binding site of complex I, NDUFV3L resembles the 68 kDa fragment of an atypical cadherin (Ft4) that has been reported to associate with complex I in Drosophila [63]. No evidence has been found for any interaction between any cadherin protein and mammalian complex I and, although a Drosophila homologue to NDUFV3 has been proposed previously [15], we have not been able to identify any high-scoring match sequence. Therefore, it is possible that NDUFV3L and Ft4 play similar roles in complex I.

The following are the supplementary data related to this article.

Supplementary Dataset 1 Proteome Discoverer output for the LC-MS dataset. Supplementary Dataset 1

Supplementary data tables Image 1

## Transparency document

Transparency document. Image 2

## Figures and Tables

**Table 1:** ***N*-terminal modifications and mitochondrial target peptide cleavage sites for the subunits of mammalian complex I.** Information for *B. taurus* was taken from [11], [43]. Information from rat is experimentally determined unless in square brackets, in which case it has been predicting by combining data from *B. taurus* with sequence-based prediction of the cleavage sites.

Subunit	*B. taurus*	*R. norvegicus*
ND1	Formyl	Formyl
ND2	Formyl	[Formyl]
ND3	Formyl	Formyl
ND4	Formyl	Formyl
ND5	Formyl	Formyl
ND6	Formyl	[Formyl]
ND4L	Formyl	Formyl
NDUFA1	no mod	[no mod]
NDUFA10	∆ 1–23	∆ 1–35
NDUFA11	-Met + Acetyl	[− Met + Acetyl]
NDUFA12	+ Acetyl	+ Acetyl
NDUFA13	-Met + Acetyl	-Met + Acetyl
NDUFA2	-Met + Acetyl	-Met + Acetyl
NDUFA3	-Met + Acetyl	[− Met + Acetyl]
NDUFA5	-Met + Acetyl	[− Met + Acetyl]
NDUFA6	-Met + Acetyl	-Met + Acetyl
NDUFA7	-Met + Acetyl	-Met + Acetyl
NDUFA8	-Met	-Met
NDUFA9	∆ 1–35	[∆ 1–35]
NDUFAB1	∆ 1–68	∆ 1–68
NDUFB1	-Met	-Met (partial)
NDUFB10	-Met	-Met
NDUFB11	∆ 1–29	∆ 1–29
NDUFB2	∆ 1–36	∆ 1–33
NDUFB3	-Met + Acetyl (partial)	-Met + Acetyl (partial)
NDUFB4	-Met + Acetyl	-Met + Acetyl
NDUFB5	∆ 1–46	[∆ 1–46]
NDUFB6	-Met + Acetyl	-Met + Acetyl
NDUFB7	-Met + Myristoyl	-Met + Myristoyl
NDUFB8	∆ 1–28	[∆ 1–28]
NDUFB9	-Met + Acetyl	-Met + Acetyl
NDUFC1	∆ 1–27	[∆ 1–27]
NDUFC2	+ Acetyl	+ Acetyl
NDUFS1	∆ 1–23	∆ 1–23
NDUFS2	∆ 1–33	[∆ 1–33]
NDUFS3	∆ 1–38	[∆ 1–36]
NDUFS4	∆ 1–42	∆ 1–42
NDUFS5	-Met	-Met
NDUFS6	∆ 1–28	[∆ 1–20]
NDUFS7	∆ 1–37	∆ 1–37
NDUFS8	∆ 1–36	∆ 1–34
NDUFV1	∆ 1–20	∆ 1–20
NDUFV2	∆ 1–32	∆ 1–31
NDUFV3	∆ 1–34	∆ 1–35

**Table 2:** **Summary of mass spectrometry evidence for the long and short NDUFV3 isoforms.** Rat samples purified by chromatography (C) or immuno-purification (IP) were analyzed by LC-MS and MALDI-TOF mass spectrometry. Peptides from the human cell line U2OS are those found in the gel slice containing intact complex I in the blue native PAGE analysis. Mascot scores for each identification are presented, compared to the *p* < 0.05 ions score cut-off for each experiment.

		Number peptides observed			Score	
	Sample	Long	Short	Common	Long	Short
Heart	LC-MS (IP)	2	3	5	731/22	720/22
	LC-MS (C)	0	1	2	1761/22	2034/22
	MALDI (IP)	0	0	0	0	0
	MALDI (C)	0	0	0	0	0
Skeletal muscle	LC-MS (IP)	2	3	5	1397/22	1554/22
	LC-MS (C)	0	2	2	1438/22	1620/22
	MALDI (IP)	0	0	0	0	0
	MALDI (C)	0	0	0	0	0
Liver	LC-MS (IP)	5	2	2	580/22	423/22
	LC-MS (C)	3	1	2	220/22	222/22
	MALDI (IP)	0	0	0	0	0
	MALDI (C)	0	0	0	0	0
Kidney	LC-MS (IP)	1	3	3	781/21	978/21
	LC-MS (C)	0	0	1	33/22	33/22
	MALDI (IP)	0	0	0	0	0
	MALDI (C)	0	0	0	0	0
Brain	LC-MS (IP)	18	2	2	1211/22	318/22
	LC-MS (C)	5	2	2	702/22	457/22
	MALDI (IP)	0	0	0	0	0
	MALDI (C)	1	0	0	42/34	0
MH-TC-5123	LC-MS (IP)	39	4	0	2448/23	109/23
cell line	MALDI (IP)	3	0	0	228/31	0
U2OS cell line	LC-MS (complexome)	10	1	0	748/25	109/25
