# Supplementary material for: Subunit NDUFV3 is present in two distinct isoforms in mammalian complex I
Source: Biochim Biophys Acta. 2017 Mar;1858(3):197–207. doi: 10.1016/j.bbabio.2016.12.001 (PMC5293009; doi:10.1016/j.bbabio.2016.12.001)
Supplement: Supplementary file 2 — Supplementary data tables [file mmc2.pdf]

SUPPLEMENTARY INFORMATION

Subunit NDUFV3 is present in two distinct isoforms in mammalian complex I

Hannah R. Bridges, Khairunnisa Mohammed, Michael E. Harbour and Judy Hirst

Table S1

Subunit/ protein	Heart			Skeletal muscle			Brain			Kidney			Liver		
	score /22	cov.	pep.	score /22	cov.	pep.	score /22	cov.	pep.	score /22	cov.	pep.	score /22	cov.	pep.
ND1	506	23%	8	518	23%	9	334	15%	6	349	20%	7	260	14%	5
ND2	373	12%	2	162	7%	1	179	7%	1	46	7%	1			
ND3				41	29%	1				58	29%	1	74	7%	1
ND4	1184	17%	9	959	17%	7	808	19%	7	823	17%	8	258	19%	6
ND5	1862	24%	9	2229	25%	11	918	14%	7	795	20%	8	368	12%	6
ND6															
ND4L	39	23%	1							79	23%	1			
NDUFA1	217	14%	1	205	14%	1	77	14%	1	38	14%	1	56	14%	1
NDUFA10	4289	64%	24	3555	66%	23	2315	61%	20	274	48%	11	1087	53%	16
NDUFA11	427	11%	4	253	11%	3	111	10%	2				143	11%	3
NDUFA12	1691	67%	11	1282	66%	12	1227	62%	10	277	33%	5	584	62%	9
NDUFA13	3435	85%	17	3358	82%	13	1904	63%	10	1408	66%	10	988	78%	12
NDUFA2	625	36%	5	786	43%	6	416	42%	5	151	34%	3	334	36%	4
NDUFA3	306	86%	3	199	86%	3	84	29%	1	88	57%	2	85	13%	1
NDUFA5	2153	65%	12	2366	73%	14	1282	59%	9	1488	55%	11	693	65%	12
NDUFA6	791	49%	7	779	49%	7	419	47%	6	285	32%	4	384	47%	6
NDUFA7	813	61%	8	1066	51%	9	615	49%	8	258	21%	3	338	42%	7
NDUFA8	850	64%	8	1066	15%	3	405	27%	5	176	18%	3	132	18%	3
NDUFA9	13075	76%	27	8610	74%	28	3377	69%	20	1170	60%	18	1430	69%	21
NDUFAB1	878	43%	7	695	43%	7	603	34%	4	34	19%	2	209	43%	5
NDUFB1	984	86%	8	670	86%	8	487	70%	5	395	84%	7	201	68%	4
NDUFB10	2789	65%	14	3041	65%	14	1275	48%	11	1231	47%	10	694	53%	11
NDUFB11	924	63%	7	1334	89%	9	529	61%	6	188	8%	1	253	59%	5
NDUFB2	286	97%	4	219	97%	4	104	26%	1	151	67%	3	157	46%	2
NDUFB3	1241	63%	8	1337	77%	10	387	35%	3	923	62%	9	433	43%	4
NDUFB4	1119	68%	11	1071	75%	11	628	55%	5	714	40%	5	355	55%	6
NDUFB5	1420	44%	10	1428	73%	10	693	42%	7	320	25%	4	490	46%	8
NDUFB6	2347	73%	11	2266	73%	10	1397	61%	9	837	55%	9	344	38%	5
NDUFB7	11229	45%	7	8871	34%	5	3614	45%	5	178	35%	3	759	45%	6
NDUFB8	3046	52%	14	3446	60%	14	2295	51%	12	268	36%	8	573	49%	11
NDUFB9	1367	46%	8	1745	45%	7	1085	45%	6	114	25%	4	223	48%	6
NDUFC1	86	78%	2	185	78%	2	28	22%	1	182	78%	3			
NDUFC2	1072	66%	10	875	56%	9	556	41%	7	565	51%	9	417	35%	6
NDUFS1	31029	63%	40	26827	63%	42	12267	53%	32	1692	33%	18	3172	51%	33
NDUFS2	23441	79%	28	18833	80%	28	4979	75%	24	1609	39%	12	2030	60%	19
NDUFS3	6860	61%	16	6020	62%	17	2185	60%	14	1243	59%	12	1425	74%	16
NDUFS4	1697	68%	12	2339	69%	15	841	68%	11	277	26%	4	594	47%	8
NDUFS5	555	40%	4	476	39%	4	344	32%	3	66	28%	3	143	19%	2
NDUFS6	995	70%	7	822	54%	6	693	50%	5	153	48%	4	315	50%	6
NDUFS7	1846	31%	7	2133	49%	9	700	30%	6	247	17%	3	531	30%	6
NDUFS8	2864	41%	8	2005	61%	9	1604	39%	6	1003	26%	6	564	46%	9
NDUFV1	4801	50%	22	4346	38%	19	2118	38%	17	904	32%	13	742	38%	17
NDUFV2	3159	75%	13	3686	75%	15	1874	51%	9	1393	39%	7	759	46%	8
NDUFV3L	1761	7%	2	1438	7%	2	702	28%	7	33	2%	1	220	27%	5
NDUFV3S	2034	71%	3	1620	71%	4	458	71%	4	33	14%	1	223	71%	3
ACAD9	223	11%	7	90	7%	3	90	7%	3	53	3%	2	348	14%	7
AIF1	153	9%	4	59	5%	2	59	5%	2	25	2%	1	23	2%	1
ECSIT	121	10%	4	108	6%	2	108	6%	2				35	5%	1
FOXRED1	39	2%	1							44	2%	1			
lactB	221	15%	6	200	13%	6	200	13%	6	251	17%	71	1197	35%	16
NDUFAF3	64	6%	1	50	6%	1	50	6%	1						
NDUFAF4	94	19%	2	63	8%	1	63	8%	1						
TMEM126A	221	36%	6	144	32%	4	144	32%	4						
TMEM261	33	13%	1	42	13%	1	49	13%	1						

Subunit identification of chromatographically-purified complexes I by LC-MS analyses. Subunit coverage (cov.) has been calculated using the mature protein lengths in Table 1. Mascot scores for each identification are presented, compared to the $p < 0.05$ ions score cut-off for each experiment. pep., the number of peptides detected.

Table S2

Subunit/ protein	Heart IP			Skeletal muscle IP			Brain IP			Kidney IP			Liver IP			MH-TC-5123		
	score /22	cov.	pep.	score /22	cov.	pep.	score /22	cov.	pep.	score /22	cov.	pep.	score /22	cov.	pep.	score /22	cov.	pep.
ND1	576	15%	6	542	16%	8	485	15%	7	491	15%	6	142	9%	3			
ND2	151	5%	1	659	12%	2	62	7%	1	230	12%	2						
ND3				63	13%	1	80	13%	1	51	13%	1						
ND4	543	19%	6	791	19%	7	938	19%	7	1134	19%	7	142	13%	3			
ND5	1448	14%	7	1549	17%	10	1502	17%	9	1922	21%	10	349	12%	6	62	2%	1
ND6																		
ND4L																		
NDUFA1	209	14%	1	124	17%	2	235	14%	1	214	17%	2	181	14%	1			
NDUFA10	1706	52%	21	3521	70%	30	3373	70%	29	3931	71%	31	1768	57%	19	64	14%	3
NDUFA11	400	11%	4	367	11%	4	437	38%	6	449	22%	5	106	11%	2			
NDUFA12	1683	71%	18	2267	79%	21	2704	86%	21	3073	73%	21	1622	70%	14	420	41%	7
NDUFA13	4158	72%	18	5564	75%	23	4523	72%	17	4293	72%	18	2017	68%	13	1457	71%	15
NDUFA2	678	36%	5	615	43%	8	385	36%	5	556	58%	7	581	36%	4	562	77%	10
NDUFA3	175	42%	2	118	42%	2	178	42%	2	158	42%	2	195	13%	1	37	13%	1
NDUFA5	1907	91%	19	2734	91%	22	1424	88%	18	2398	90%	19	832	64%	8	164	35%	3
NDUFA6	1702	55%	10	1482	72%	15	1189	55%	9	1034	55%	8	876	55%	8	259	67%	8
NDUFA7	1653	70%	9	2509	83%	16	1554	75%	12	1751	79%	13	782	60%	10	1827	91%	23
NDUFA8	948	60%	9	1009	48%	8	674	46%	7	847	59%	8	416	40%	5	144	34%	3
NDUFA9	2777	73%	24	3697	72%	28	4569	76%	26	5952	73%	24	1954	68%	22	203	28%	5
NDUFAB1	721	43%	7	643	43%	7	656	36%	6	818	43%	7	700	36%	6			
NDUFB1	401	100%	7	802	100%	9	287	51%	5	378	86%	7	87	47%	3	131	63%	4
NDUFB10	2191	72%	17	2292	66%	14	2402	72%	17	2154	72%	16	1616	57%	12	511	16%	3
NDUFB11	1046	64%	8	1212	66%	10	1095	63%	8	1159	64%	8	261	63%	6	348	55%	6
NDUFB2	190	60%	3	130	60%	3	171	60%	3	135	40%	2	28	14%	1	25	14%	1
NDUFB3	1173	47%	7	1464	62%	9	879	47%	7	1051	47%	7	550	47%	6	368	26%	7
NDUFB4	579	48%	8	1150	81%	14	1076	55%	10	1002	57%	11	822	54%	8	447	64%	9
NDUFB5	1458	45%	11	1727	46%	15	1689	45%	11	1881	45%	13	938	44%	10	295	38%	8
NDUFB6	1732	66%	13	2056	66%	14	2298	66%	14	1952	83%	13	1153	59%	9	356	40%	7
NDUFB7	3270	54%	12	2968	47%	11	4489	47%	11	4762	47%	10	950	47%	8	94	12%	1
NDUFB8	3013	59%	20	4315	64%	27	3151	59%	19	3593	59%	21	1718	51%	13	371	40%	10
NDUFB9	1452	56%	12	2463	68%	19	1910	63%	12	2238	63%	14	981	54%	8	154	31%	4
NDUFC1	102	22%	1	106	78%	2	88	22%	1	76	31%	2	92	22%	1			
NDUFC2	1093	61%	12	1291	60%	13	978	61%	10	1407	61%	12	812	60%	10	554	44%	6
NDUFS1	9779	66%	48	14786	73%	67	14868	72%	55	17263	73%	56	5200	53%	32	1494	40%	33
NDUFS2	7399	72%	29	10983	70%	32	9438	74%	32	12585	77%	35	2785	59%	23	119	12%	5
NDUFS3	3308	61%	22	3321	66%	27	2904	61%	22	3651	74%	24	1978	55%	15	507	42%	9
NDUFS4	1240	70%	12	2043	84%	19	1174	71%	15	1552	70%	14	808	69%	11	507	64%	13
NDUFS5	969	43%	5	742	46%	9	775	58%	9	807	46%	8	377	33%	3	229	26%	4
NDUFS6	1660	75%	10	1730	75%	10	1174	75%	11	1284	59%	9	768	71%	9	1765	65%	10
NDUFS7	1312	49%	9	1819	58%	13	2011	55%	13	2212	49%	13	711	44%	6	167	14%	3
NDUFS8	1331	46%	9	4950	55%	17	3160	46%	11	4335	48%	12	963	41%	8	276	27%	4
NDUFV1	2756	49%	27	2920	59%	38	3227	65%	33	3092	47%	28	1742	42%	22	224	17%	8
NDUFV2	3546	53%	15	2539	73%	19	2470	70%	14	3087	56%	14	1796	44%	10	266	35%	7
NDUFV3L	731	38%	11	1397	20%	7	1212	74%	20	781	10%	4	580	23%	7	2448	66%	39
NDUFV3S	720	82%	6	1554	81%	8	318	71%	4	978	81%	6	423	71%	4	109	64%	4
ACAD9	31	1%	1	24	1%	1	72	4%	3	237	12%	8	49	1%	1			
ECSIT							30	2%	1	82	8%	3						
Isu1										51	7%	1						
LYRM1	164	27%	4	119	24%	4	177	34%	5	205	29%	4	88	20%	2			
LYRM2	171	45%	5				96	31%	3	147	41%	5	78	31%	3			
LYRM4	418	66%	9	555	73%	11	472	69%	10	626	69%	10	546	66%	8	146	40%	4
LYRM5	139	43%	6	338	65%	9				310	65%	9	244	73%	8			
LYRM7	349	28%	2	291	38%	3	263	28%	2	302	28%	2	76	10%	1	59	10%	1
LYRM9	385	54%	6	212	56%	7	322	62%	6	217	54%	4	160	32%	2			
NDUFAF1							24	2%	1	58	5%	1						
NDUFAF4										28	10%	1						
Nfs1	485	33%	12	593	32%	13	1992	52%	20	3495	50%	24	1346	41%	16	136	22%	8
TMEM126A					21%	3				158	27%	5						
TMEM261										43	13%	1	27	13%	1			

Subunit identification of immuno-purified complexes I by LC-MS analyses. Subunit coverage (cov.) has been calculated using the mature protein lengths in Table 1. Mascot scores for each identification are presented, compared to the $p < 0.05$ ions score cut-off for each experiment. pep., the number of peptides detected.

Table S3

Subunit	Modification	Experimental peptide mass	Calculated peptide mass	Δmass (ppm)	Ions score /22	Peptide
ND1	Formyl	3076.7872	3076.7836	1.17	62	MYFINILTLLIPILIAMAFLTLVERK + Formyl (*N*-term)
ND2	Formyl	-	-	-	-	-
ND3	Formyl	3893.1472	3893.149	-0.45	54	MNLLIIITINITLSFILISIAFWLPQMNLYSEK + Formyl (*N*-term)
ND4	Formyl	2456.3928	2456.3939	-0.44	66	MLKIIFPSIMLLPLTWLSANK + Formyl (*N*-term)
ND5	Formyl	3176.7013	3176.7006	0.21	72	MNMMTSSILMILILLTTPIIISMTNLPK + Oxidation (Met) + Formyl (*N*-term)
ND6	Formyl	-	-	-	-	-
ND4L	Formyl	2640.3056	2640.3154	-3.73	71	MTSAFLNLTMAFTLSLLGTFMFR + Formyl (*N*-term)
NDUFA1	-	-	-	-	-	-
NDUFA10	Δ1-35	1417.8298	1417.8293	0.4	56	LRYGLLASILGDK
NDUFA11	-Met + Acetyl	1584.8587	1584.8583	0.25	34	ESSRAVIAPSGVERK
NDUFA12	+ Acetyl	1057.5958	1057.5954	0.42	51	MEQLRVLK + Acetyl (*N*-term)
NDUFA13	-Met + Acetyl	2416.2199	2416.2209	-0.39	52	AASKVKQDMPPPGGYGPIDYKR + Acetyl (*N*-term)
NDUFA2	-Met +Acetyl	970.5558	970.556	-0.19	73	AAASRVVGAK + Acetyl (*N*-term)
NDUFA3	-Met + Acetyl	-	-	-	-	-
NDUFA5	-Met + Acetyl	-	-	-	-	-
NDUFA6	-Met + Acetyl	2359.2648	2359.2608	1.73	51	AAAASGLRQAASAASTSVKPIFSR + Acetyl (*N*-term)
NDUFA7	-Met + Acetyl	1014.5829	1014.5822	0.75	42	ASATRVIQK + Acetyl (*N*-term)
NDUFA8	-Met	1436.8119	1436.8126	-0.52	88	PGIVELPTLEELK
NDUFA9	Δ1-35	-	-	-	-	-
NDUFAB1	Δ1-68	1267.6773	1267.6772	0.12	75	SDAPPLTLEGIR
NDUFB1	-Met (partial)	972.5804	972.579	1.46	68	MTLLQLVR
		841.5394	841.5385	1.05	51	TLLQLVR
NDUFB10	-Met	1699.7837	1699.7842	-0.31	92	PDSWDKDVYPEPPR
NDUFB11	Δ1-29	-	-	-	-	-
NDUFB2	Δ1-33	1034.5257	1034.5258	-0.028	76	VGDGGHIQPR
NDUFB3	-Met + Acetyl (partial)	2292.0368	2292.0355	0.57	49	AAGHGHEHGHGHGKMELPDYR
		2334.047	2334.0461	0.41	36	AAGHGHEHGHGHGKMELPDYR + Acetyl (*N*-term)
NDUFB4	-Met + Acetyl	3218.6226	3218.6135	2.82	73	SFSKYKPAPLAALPNTLDPAEYDVSPETR + Acetyl (*N*-term)
NDUFB5	Δ1-46	-	-	-	-	-
NDUFB6	-Met + Acetyl	1206.5894	1206.588	1.16	65	SGYTPDEKLR + Acetyl (*N*-term)
NDUFB7	-Met + Myristoyl	863.5606	863.5593	1.6	50	GAHLTR + Myristoyl (*N*-term)
NDUFB8	Δ1-28	-	-	-	-	-
NDUFB9	-Met + Acetyl	1387.6716	1387.6707	0.68	61	AFCAPPAYLTHR + Acetyl (*N*-term)
NDUFC1	Δ1-27	-	-	-	-	-
NDUFC2	+ Acetyl	1435.6809	1435.6812	-0.25	37	MMNGRPGHEPLR + Acetyl (*N*-term)
NDUFS1	Δ1-23	1435.7198	1435.7195	0.24	39	TGTAASNLIEVFVD
NDUFS2	Δ1-33	-	-	-	-	-
NDUFS3	Δ1-36	-	-	-	-	-
NDUFS4	Δ1-42	1788.8845	1788.8854	-0.47	31	ADGQTRDTQLITVDEK
NDUFS5	-Met	1001.5664	1001.5658	0.58	47	PFLDVQKR
NDUFS6	Δ1-20	-	-	-	-	-
NDUFS7	Δ1-37	1899.9334	1899.9286	2.53	113	AHQSVAATGSPSSTQSAVSK
NDUFS8	Δ1-34	2075.0055	2075.0067	-0.58	57	TYKYVNMKAQELDVDMK
NDUFV1	Δ1-20	3075.5637	3075.5625	0.37	25	SSGTTAPKKTSFGSLKDEDRIFTNLYGR
NDUFV2	Δ1-31	983.5291	983.5301	-0.97	80	GAGGALFVHR
NDUFV3	Δ1-35	3987.9462	3987.9436	0.64	32	STESENNKKAAGPTSKTESVFKEPTLVPESSDTTTYK

Peptide evidence for the modifications of *N*-terminal peptides.

Table S4

	Peptide	2^+	3^+	4^+
Long	ESTELFEAEGILPGHR	X	X	
	VSTQPTTGTQEASAELRPAAAPESGAR	X		
Common	FRLPQPSSGR	X		
	NLQHHEYNAFTFLDLNLDLSK	X	X	X
Short	TESVFKEPTLVPESSDTTTYK	X	X	
	EPTLVPESSDTTTYK	X		

Peptides selected for relative quantification. The selected peptides do not contain methionine or cysteine residues that are subject to variable modifications. The scarcity of unique peptides in the short 10 kDa isoform necessitated the selection of two peptides with overlapping sequences, one of which results from a missed tryptic cleavage. The charge states of ions identified in at least one sample are shown.
